# 

**Published:** 1892-08-20

**Authors:** 


CONTENTS.

PAGE
**Asylum or Hospital?** . . . . 337
**Passing Topics.**—House Governor or Medical Superintendent?—II. . . . . 338
**The Doctor's Armchair.**—The Collapse of Matteism . . . 339
**The Attitude of Great Writers towards Disease.** X.—Keats and Shelley . . . . 340
**Medical History from the Earliest Times.** By E. T. WITHINGTON, M.A., M.B.Oxon. XXII.—Official Medicine in Ancient Rome . . . . 341
**Editor's Letter-Box.**—Royal Hospital for Incurables—Hospital Saturday Fund . . . . 342
**Annotations.**—Sewing in Schools—A Question for Mr. Keir Hardie—Medical Sickness and Annuity . . . . 343
**Notes and News** . . . . 344
**Scraps and Gleanings** . . . . 345
**The Practitioners' Mirror and Retrospect—**
MEDICINE.—The Treatment of Sleeplessness Complicating other Diseases, 346;—The Medical Treatment of Perityphlitis and Appendicitis . . . . 347
THERAPEUTICS.—The Choice of Calcium Salts . . . . 348
**The Institutional Workshop—**
HOSPITAL CONSTRUCTION.—Ladywell Sanatorium, Salford . 349
HOSPITAL OPINION.—The Asylum Attendant of To-day, 351; Hospital Administration: Finance . . . . 352
**The Student of Medicine.** — Appointments—Vacancies—Pass Lists.

EXTRA SUPPLEMENT.—THE NURSING MIRROR.

**En Passant.**—The Aberdeen District Nursing Association—Lady Students at the Edinburgh Royal Infirmary—Not Lost Sight of—Isle of Man Nurses—District Nurses and the Poor Law—"Manners Maketh Man" . . . . cxlv

**The Management of Consumptive Patients.** III.—General Advice . . . . cxlvi

**Death in Our Ranks** . . . . cxlvi

**The Nightingale Fund.**—Report for 1891 . . . . cxlvii

**Nursing Homes.**—Cambridge Home and Training School for Nurses.—VI . . . . cxlviii

**For Reading to the Sick.**—Real Kindness . . . . cxlvii

**Why Probationers Break Down** . . . . cxlviii

**Nurses' Bookshelf.**—"The Naulahka"—Essentials of Medical Electricity . . . . cxlix

**Some Thoughts about Holidays** . . . . cl

**Appointments** . . . . cl

**The Little Sisters of the Assumption** . . . . cli

**Royal Berks Hospital, Reading** . . . . cli

**Off Duty.**—A Long Day . . . . clii

**Notes and Queries** . . . . clii

